# Angiopoietin-1 derived peptide hydrogel promotes molecular hallmarks of regeneration and wound healing in dermal fibroblasts

**DOI:** 10.1016/j.isci.2023.105984

**Published:** 2023-01-14

**Authors:** Katrina Vizely, Karl T. Wagner, Serena Mandla, Dakota Gustafson, Jason E. Fish, Milica Radisic

**Affiliations:** 1 Department of Chemical Engineering and Applied Chemistry, University of Toronto, Toronto, ON M5S 3E5, Canada; 2 Institute of Biomaterials and Biomedical Engineering, University of Toronto, Toronto, ON M5S 3G9, Canada; 3 Toronto General Hospital Research Institute, University Health Network, Toronto,ON M5G 2C4, Canada; 4 Department of Laboratory Medicine and Pathobiology, University of Toronto, Toronto, ON M5S 1A8, Canada

**Keywords:** Biomaterials, Health sciences, Materials science

## Abstract

By providing an ideal environment for healing, biomaterials can be designed to facilitate and encourage wound regeneration. As the wound healing process is complex, there needs to be consideration for the cell types playing major roles, such as fibroblasts. As a major cell type in the dermis, fibroblasts have a large impact on the processes and outcomes of wound healing. Prevopisly, conjugating the angiopoietin-1 derived Q-peptide (QHREDGS) to a collagen-chitosan hydrogel created a biomaterial with *in vivo* success in accelerating wound healing. This study utilized solvent cast Q-peptide conjugated collagen-chitosan seeded with fibroblast monolayers to investigate the direct impact of the material on this major cell type. After 24 h, fibroblasts had a significant change in release of anti-inflammatory, pro-healing, and ECM deposition cytokines, with demonstrated immunomodulatory effects on macrophages and upregulated expression of critical wound healing genes.

## Highlights

• QHREDGS peptide hydrogel supports dermal fibroblasts without excess proliferation
• Fibroblasts on Q-peptide uniquely secrete both pro- and anti-inflammatory cytokines
• Anti-fibrotic cytokines are secreted alongside those required for ECM deposition
• Fibroblasts on Q-peptide exhibit upregulation of 15 critical wound healing genes

## Graphical abstract

**Figure:**
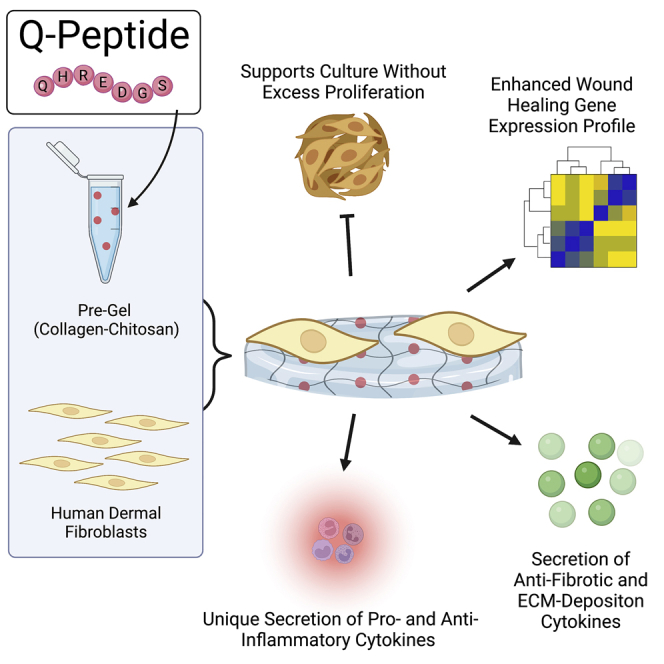

## Introduction

As the largest organ, the skin has a monumental task; it provides a barrier against the external world. This critical structure endures mechanical damage, ultraviolent radiation, extreme temperatures and microbial presence to maintain the ideal conditions for the internal organs to function.^1^^,^^2^^,^^3^ The importance of skin translates into a large cost associated with injury and healing. In the US alone, non-healing wounds, scarring, and burns present large problems for the patient and the medical system, accounting for $50, $12, and $7.5 billion respectively every year.^4^ Certain populations are especially predisposed to aberrant healing, compounding their medical problems.^2^ Despite the need, advancing would healing therapies to the clinic has proven to be extremely difficult^5^^,^^6^

Traditional approaches to wound healing have diverged into two strategies: (1) Providing the optimal environment for wound healing and (2) modulating cellular processes to initiate the closure of a wound. Wound dressings that have focused on providing the optimal healing environment for the native cellular processes, are inherently effective yet limited. They cannot completely account for the dysregulated cellular processes that cause slow or lack of wound closure, and excessive scar tissue formation.^5^ Attempts to influence the wound healing process with the addition of growth hormones provided wound closure at the expense of increased cancer, highlighting a challenge in engineering biomaterials safely.^7^ To alleviate this medical dilemma, biomaterial development is necessary to provide the optimal macroscopic environment while safely modulating the wound healing process to provide wound closure and scar attenuation.^8^

An example of a biomaterial with an observed impact on wound healing is a peptide conjugated collagen chitosan hydrogel. Previous studies have highlighted the profound impact of the angiopoiten-1 derived peptide, QHREDGS (Q-peptide), in the wound closure of a diabetic mouse,^9^ an equine model,^10^ human split thickness grafts^11^ and supporting the survival of cardiomyocytes.^12^ The Q-peptide has been chemically conjugated to a collagen-chitosan hydrogel to maintain a stable wet healing environment, with demonstrated accelerated keratinocyte migration and immune modulation.^13^ Despite the observed functional efficacy of this peptide biomaterial, the direct and indirect mechanisms for the pro-healing response in wounds have not been fully characterized.

The skin is a layered organ with two main sections: the epidermis and the dermis. These layers rely on keratinocytes and fibroblasts, respectively, to maintain a barrier and provide the mechanical properties of the skin.^3^ Naturally, as these properties are integral for wound healing, they are integral to designing biomaterials for wound healing applications.^14^ The epidermis is primarily populated with specialized epithelial cells, keratinocytes. Other cells such as Langerhans cells, melanocytes and Merkel cells are responsible for immune regulation, pigmentation and sensory function, leaving keratinocytes to provide and maintain the barrier functionality of skin.^15^^,^^16^ Below the epidermis is the dermis, where the primary cell population is the dermal fibroblasts. These cells are responsible for the synthesis and remodeling of extracellular proteins. In addition to producing structurally necessary proteins, fibroblasts also participate in complex cellular signaling processes involving keratinocytes, immune cells, endothelial cells, and mast cells.^17^

As a ubiquitous cell within connective tissue of every organ, fibroblasts deposit and remodel ECM. In wound healing, this role is very important to maintain the integrity of the barrier that skin provides. Fibroblast heterogeneity can have implications in the phenotype of these cells and even variable functions in wound healing such as ECM deposition and organization, secretion of growth factors and cytokines and immune modulation.^1^ Fibroblasts from chronic, non-healing wounds often display an atypical phenotype which may include decreased proliferation, early senescence, and altered patterns of cytokines release.^18^ This contrasts with fibroblasts from keloid scars where proliferation is accelerated, and apoptosis has decreased.^19^

The release of cytokines is critical for healthy wound healing as cellular coordination is integral for prevention of fibrosis and scarring. As a cell type, fibroblasts are quite plastic and very responsive to signals from the epidermis and other cells within the dermis.^1^ As the phenotype of fibroblasts can greatly impact wound healing it is an ideal target for testing the impact of Q-peptide, to observe whether a beneficial response is initiated.

Cytokine release from the cells within a wound is integral for normal healing through appropriate communication and recruitment.^20^^,^^21^ Aberrant signaling can lead to hypertrophic scar formation^2^ and poor wound closure. Furthermore, keratinocytes and fibroblasts not only signal each other, they also engage in signaling loops that lead to the successful closure and tissue remodeling.^22^^,^^23^ In addition to the complex and cyclic relationship between keratinocytes and fibroblasts, the ability to recruit and activate immune cells is another important aspect of wound healing signaling.^24^ The analysis of these cytokines in keratinocytes and fibroblasts to assess how they are signaling each other and the potential for immune recruitment will be a necessary part of assessing the impact of Q-peptide hydrogels on wound healing and scar reduction.

The Q-peptide hydrogels had success in animal models,^9^^,^^10^^,^^11^ and with supporting *in vitro* work^12^^,^^13^ this therapeutic is on its way to the clinic. QHREDGS peptide hydrogels may be important for recruiting fibroblasts for wound repair, creating a coordinated pro-healing, anti-inflammatory cellular response that also attenuates myofibroblast activation and fibrosis.

In this work, we assessed the impact of Q-peptide modified hydrogel on fibroblasts after one and seven days, time points consistent with healing of acute wounds. We utilized fluorescent microscopy to study the impact on cytoskeletal organization and ECM deposition after culturing the cells on Q-peptide hydrogel in comparison to the peptide-free hydrogel, scrambled peptide hydrogel, and tissue culture plastic controls. We then analyzed the release of cytokines from the cells to gain insight into the cellular response initiated by the Q-peptide hydrogel. In addition, we evaluated the expression of 26 genes implicated in wound healing in dermal fibroblasts cultivated on the Q-peptide hydrogel in comparison to the controls.

## Results

### Q-peptide hydrogel supports adult human dermal fibroblasts culture preventing excessive proliferation

**Figure 1:**
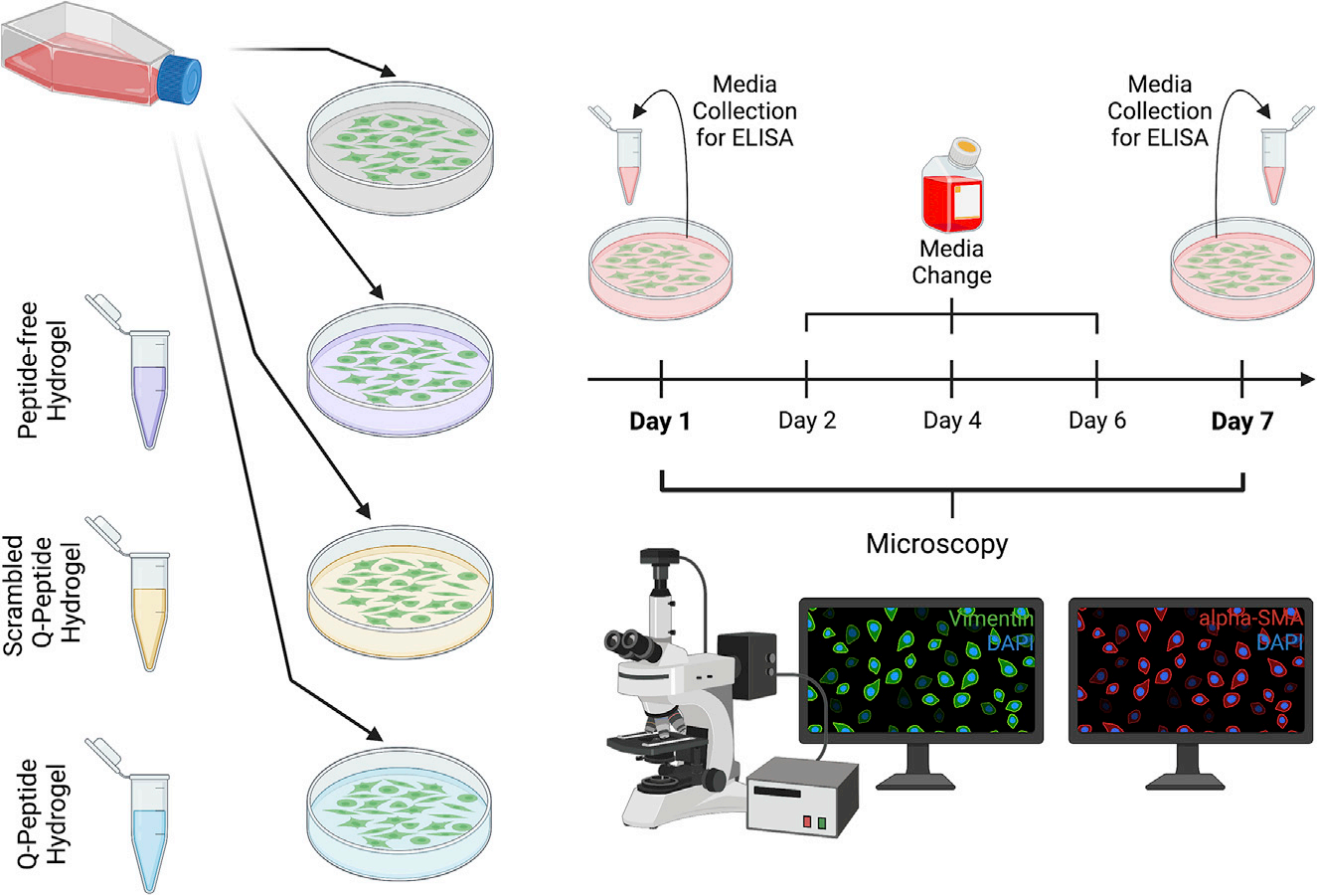
Schematic of the experimental design to study impact of Q-peptide, scrambled Q-peptide, and peptide-free hydrogel in comparison to tissue-culture plastic on normal adult human dermal fibroblasts Media is collected at Day 1 and Day 7 for Cytokine analysis via ELISA. This will allow a study of cytokines related to ECM deposition and inflammation. Fluorescent microscopy gives insight into differences in key structural proteins such as Vimentin and α−SMA at Days 1 and 7. Created with Biorender.com.

**Figure 2:**
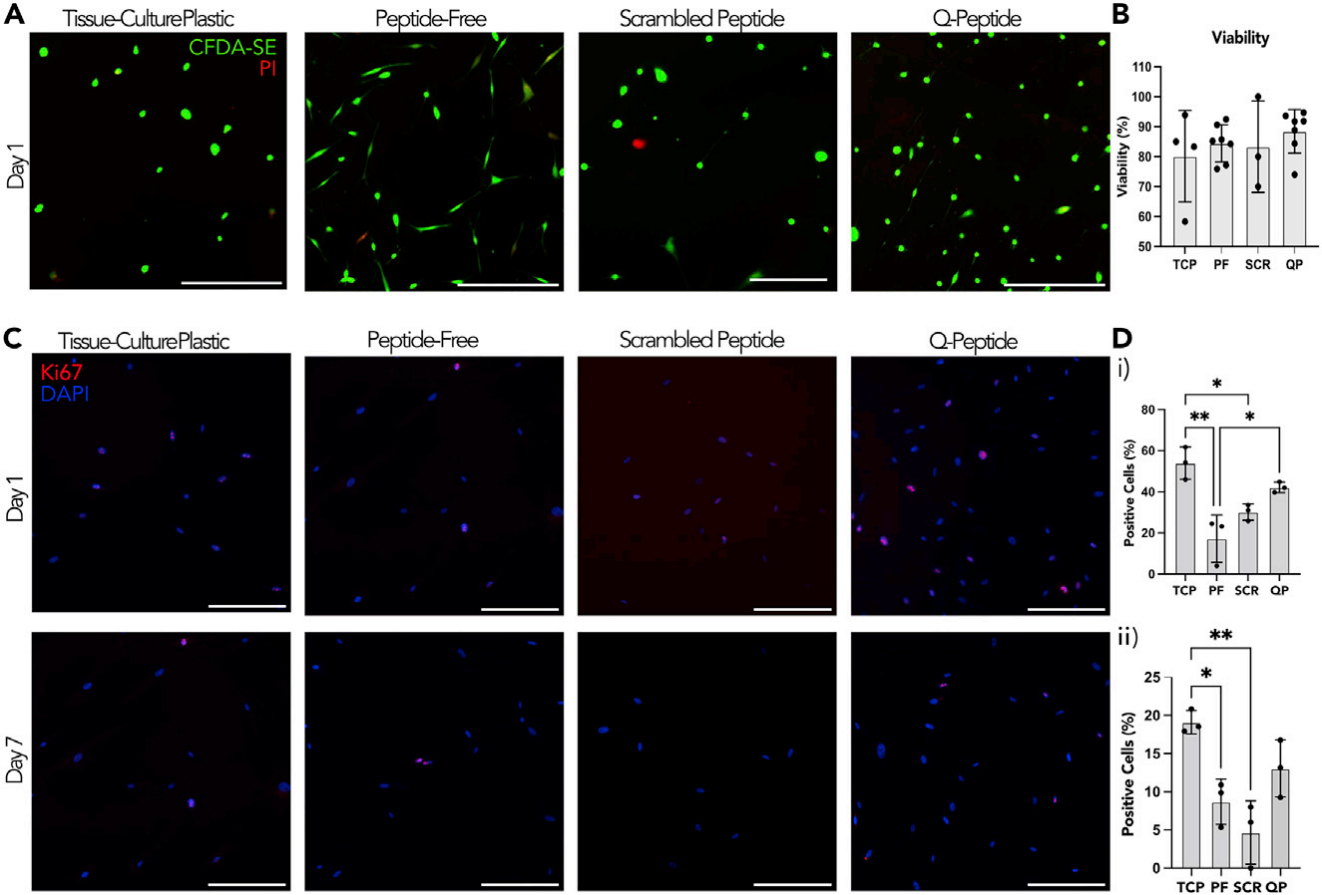
Fibroblast viability is maintained on all surfaces and proliferation is attenuated on hydrogels (A) Live-Dead staining of HDF cells after 24 h using CFDA-SE and PI (scale bars = 200 μm). Surfaces include: tissue-culture plastic (TCP), peptide-free hydrogel (PF), scrambled Q-peptide hydrogel (SCR), and Q-peptide hydrogel (QP). (B) Quantification of early cell viability on day 1 using the relative counts of CFDA-SE to PI. (C) Representative images of HDF cells stained with Ki-67 (red) and counterstained with DAPI (blue) after 24 h and seven days (scale bars = 200 μm). (D) Quantification of positive Ki-67 staining (%) revealed a significant difference between cells cultured on the Q-peptide hydrogel (QP) and the peptide-free hydrogel (PF) after (i) 24 h but not (ii) seven days. Statistical analysis included one-way ANOVA followed by a Tukey’s post hoc test. n = 3–4. Data presented as mean ±SD ∗ = p < 0.05, ∗∗ = p < 0.01.

To evaluate a biomaterial designed to influence the wound healing process, we must consider the major cell populations required to close and repair injured skin, in this case dermal fibroblasts. At Day 1 and Day 7 of their culture on the peptide modified biomaterial and appropriate controls, samples were taken for cytokine analysis and immunofluorescent staining and imaging (Figure 1). Live-dead staining of human dermal fibroblasts (HDFs) revealed that viability was not impacted significantly between the three culturing surfaces after 24 h (Figures 2A and 2B).

Immunostaining with the proliferation marker Ki67 demonstrated that cells grown on the tissue-culture plastic (TCP) had a significantly higher percentage of proliferating cells than those cultivated on the peptide-free hydrogel and the scrambled peptide (DGQESHR) hydrogel on both Day 1 and Day 7 of culture (Figures 2C and 2D). There were no significant differences in the percentage of proliferating cells on Q-peptide hydrogel compared to the scrambled peptide on Day 1 and Day 7 (Figures 2C and 2D). In addition, there was no significant difference in the percentage of proliferating Ki67 + cells between the Q-peptide hydrogel and the TCP control at either Day 1 or Day 7 of culture (Figures 2C and 2D).

### Normal fibroblast phenotype is promoted by the Q-peptide hydrogel

**Figure 3:**
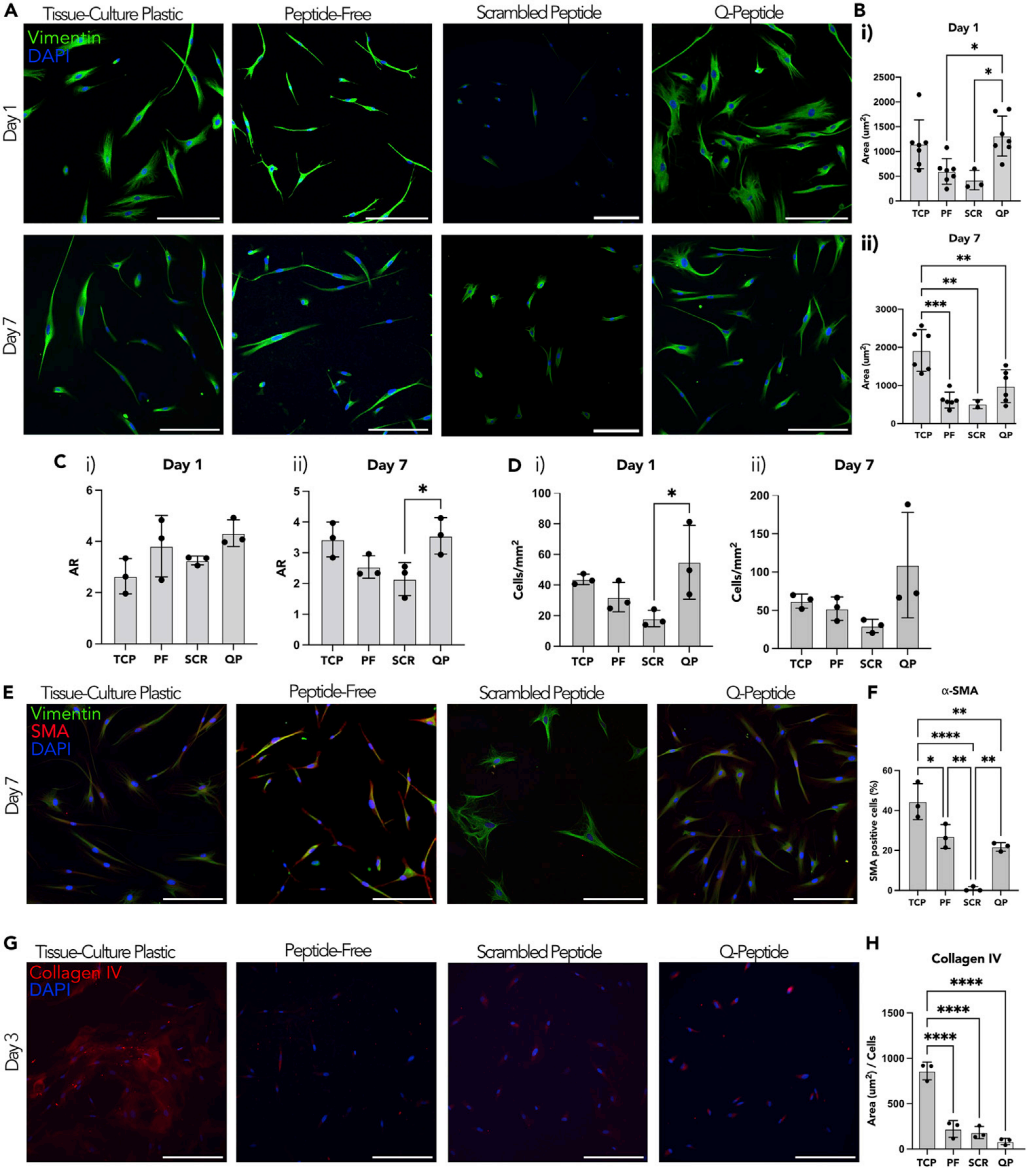
Fibroblasts express cell specific marker vimentin on all surfaces and attenuate expression of α-SMA on hydrogels (A) Comparison of HDF cells stained with Vimentin and counterstained with DAPI at days 1 and 7 (scale bars = 200 μm). Surfaces include tissue-culture plastic (TCP), peptide-free hydrogel (PF), scrambled Q-peptide hydrogel (SCR), and Q-peptide hydrogel (QP). (B) Quantification of vimentin staining revealed (i) an increase for QP relative to PF and SCR on day 1 and (ii) decreased staining for all hydrogel groups on day 7 relative to TCP. (C) Aspect Ratio (AR) quantification between the different conditions indicated (i) no significant difference in cell shape on day 1 and ii) increased AR for QP compared to SCR on day 7. (D) Cell count normalized to area (mm^2^) did not significantly differ between samples on (i) day 1 or (ii) day 7. (E) Comparison of HDF cells stained with Vimentin, α−SMA and DAPI to measure α−SMA positive cells (scale bars = 200 μm). (F) Quantification of α−SMA+ cells as a percentage of the total cell count. (G) Collagen IV staining of HDF cells on day 3 of culture (scale bars = 200 μm). (H) Quantification of collagen IV staining revealed significantly less collagen IV for all hydrogel samples when compared to tissue-culture plastic, with no significant differences between peptide-free, Q-peptide, or scrambled Q-peptide hydrogels. Statistical analysis included one-way ANOVA followed by a Tukey’s post hoc test. n = 3–4. Data presented as mean ±SD ∗ = p < 0.05, ∗∗ = p < 0.01, ∗∗∗ = p<0.001, ∗∗∗∗ = p<0.0001.

Vimentin, a major cytoskeletal component and signal integrator during wound healing in dermal fibroblasts,^25^ was immunostained and quantified using Image J (Figures 3A and 3B). On day 1, dermal fibroblasts grown on TCP and the collagen-chitosan hydrogel controls had an elongated spindle shape, typical of dermal fibroblasts; however, fibroblasts grown on the Q-peptide hydrogel appeared to be more flat and spread out (Figure 3A). In addition, on Day 1, the vimentin+ dermal fibroblasts grown on the scrambled peptide hydrogel appeared considerably less spread out than in the remaining groups (Figure 3A). Image analysis confirmed the highest cell area on Day 1 on Q-peptide hydrogel (Figure 3BI). By Day 7, spindle-shaped vimentin+ cells were visible in all groups (Figure 3A) with a significantly higher cell area on TCP substrates compared to the hydrogels (Figure 3B(ii)). Despite visual differences in cell shape and size, there was no significant difference in the aspect ratio, a ratio of the length versus width of the cells on any culture condition, from vimentin staining on Day 1 in culture (Figure 3C(i)), whereas by Day 7, aspect ratio was significantly higher on Q-peptide hydrogel compared to the controls (Figure 3C**(ii)**). Finally, cell number was significantly higher on Q-peptide hydrogel on Day 1 (Figure 3D(i)) than the other control groups, and although the trend was maintained on Day 7, the statistical significance was lost (Figure 3D**(ii)**).

The presence of α-SMA positive cells can provide insight into the rate of transition from fibroblasts to myofibroblasts. Double staining for vimentin and smooth muscle actin was used to detect the presence of myofibroblasts (Figure 3E), indicating the highest percentage on TCP substrates and almost an absence of myofibroblasts on scrambled peptide hydrogels (Figure 3F). Peptide-free and Q-peptide hydrogels presented intermediate percentages of myofibroblast presence (Figure 3E).

Collagen IV deposition was assessed by immunostaining on Day 3 of culture, indicating a significantly higher deposition on TCP compared to all other hydrogel groups (Figures 3G and 3H), likely because of the increased matrix stiffness on TCP versus softer hydrogel substrates.

### Q-peptide stimulates pro-inflammatory and anti-inflammatory cytokine release from adult human dermal fibroblasts

**Figure 4:**
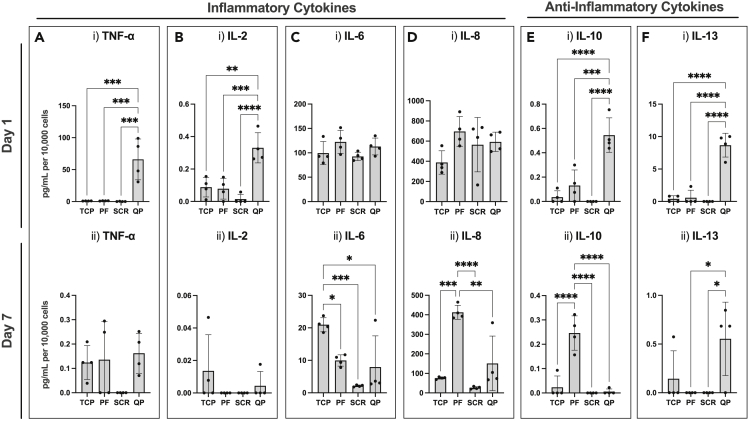
Unique secretion profile of pro- and anti-inflammatory cytokines by dermal fibroblasts cultivated on Q-peptide hydrogel Cytokine secretion of pro-inflammatory cytokines. (A–E) TNF-α, (B) IL-2, (C) IL-6, (D) IL-8, and anti-inflammatory cytokines (E) IL-10, and (F) IL-13 at (i) 1 day and (ii) 7 days of culture. Surfaces include tissue-culture plastic (TCP), peptide-free hydrogel (PF), scrambled Q-peptide hydrogel (SCR), and Q-peptide hydrogel (QP). Concentration (pg/mL) expressed after subtraction of baseline media control and normalized to cell number (10,000 cells). Statistical Analysis included One-way ANOVA followed by a Tukey’s post hoc test. n = 4. Data presented as mean ±SD ∗ = p < 0.05, ∗∗ = p < 0.01, ∗∗∗ = p<0.001, ∗∗∗∗ = p<0.0001.

Cytokine release was analyzed using ELISA to elucidate the role of the Q-peptide hydrogel on HDF phenotype. Cytokine concentrates were expressed normalized to 10,000 cells to account for slight differences in cell number over the culture period. On day 1, there was an increase in pro-inflammatory cytokines, TNF-α and interleukin (IL)-2, however this increase was not sustained by day 7 (Figures 4A and 4B), suggesting that the Q-peptide hydrogel does not lead to a sustained inflammatory state. Conversely, there was no difference in IL-6 production on day 1, and by day 7; HDFs on the Q-peptide hydrogel secreted significantly less IL-6 than the TCP control (Figure 4C). The adult HDFs cultured on the peptide-free hydrogel released a significantly higher concentration of IL-8 on day 7 compared to the TCP and the Q-peptide hydrogel (Figure 4D). Notably, secretion of inflammatory cytokines was negligible on scrambled peptide hydrogel, aside from IL-6 and IL-8 which were in line with secretion in the other groups.

In addition to pro-inflammatory cytokines, anti-inflammatory cytokines IL-10 and IL-13 were also measured. Fibroblasts cultured on the Q-peptide hydrogel released significantly more IL-10 and IL-13 on day 1 (Figure 4E(i) and 4F(i)). By day 7, HDFs cultured on the peptide-free hydrogel had sustained significantly elevated levels of IL-10 (Figure 4E(ii)), and cells on the Q-peptide hydrogel had significantly elevated levels of IL-13 (Figure 4F(ii)), though both at lower concentrations compared to day 1. Notably, nearly all cytokine concentrations were lower on day 7 compared to day 1. Importantly, secretion of anti-inflammatory cytokines on scrambled peptide controls was negligible compared to the Q-peptide hydrogels. This points to an important difference in the ability to stimulate cytokine secretion between the Q-peptide and the scrambled peptide hydrogels (Figure 4).

As the Q-peptide hydrogel is only solvent coated on the well plates and not crosslinked, an FITC-conjugated Q-peptide, was used to determine if there was any peptide release. Q-peptide release was calculated to be 86% and 64% when incubated with cells and phosphate buffered saline (PBS) only respectively for 24 h (Figure S1). This suggests the majority of the material is released within the first 24 h, resulting in the pronounced reduction in cytokines during the period, and the subdued impact by Day 7.

### Q-peptide hydrogel promotes fibroblast release of ECM producing cytokines

**Figure 5:**
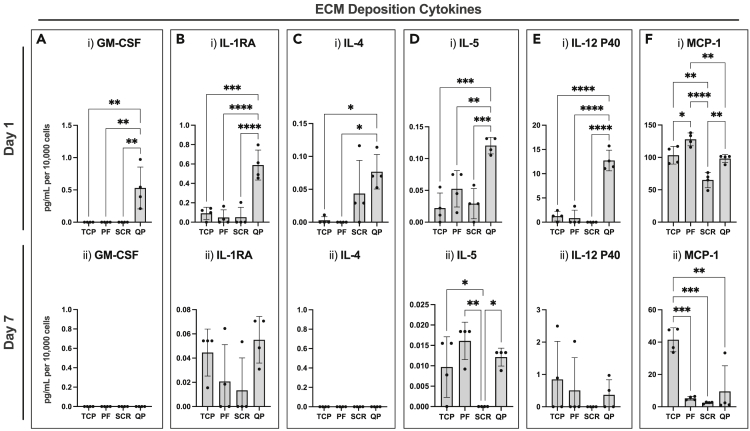
Fibroblasts cultivated on Q-peptide gel exhibit early enhanced secretion of cytokines implicated in ECM deposition (A–F) Cytokine release of (A) GM-CSF, (B) IL-1RA, (C) IL-4, (D) IL-5, (E) IL-12 P40, and (F) MCP-1 at (i) 1 day and (ii) 7 days of culture. Surfaces include tissue-culture plastic (TCP), peptide-free hydrogel (PF), scrambled Q-peptide hydrogel (SCR), and Q-peptide hydrogel (QP). Concentration (pg/mL) expressed after subtraction of baseline media control and normalized to cell number (10,000 cells). Statistical Analysis included One-way ANOVA followed by a Tukey’s post hoc test. n = 4. Data presented as mean ± SD ∗ = p < 0.05, ∗∗ = p < 0.01, ∗∗∗ = p < 0.001, ∗∗∗∗ = p < 0.0001.

Fibroblasts are a well-known contributor in the production of ECM promoting cytokines. We sought to investigate the role of the Q-peptide hydrogel on ECM cytokine production with ELISA. On day 1, GM-CSF was significantly increased in the Q-peptide hydrogel sample, however by day 7, all samples had negligible amounts of GM-CSF (Figure 5A). This trend held true for IL-1RA, IL-4, IL-5, and IL-12p40 cytokine secretion with the Q-peptide hydrogel promoting an increase in secretion on day 1 (Figures 5B–5E). Of interest, a similar trend was not observed when measuring monocyte chemoattractant protein-1 (MCP-1) release. On day 1, fibroblasts cultured on the peptide-free hydrogel secreted a significantly higher concentration of MCP-1 compared to the other culture groups, but by day 7, HDFs cultured on TCP secreted significantly higher concentrations of MCP-1 (Figure 5F). This highlights the importance of studying the temporal effect of culture conditions on fibroblast cytokine secretion. Importantly, the secretion of all cytokines illustrated in Figure 5, aside from MCP-1 was negligible on the scrambled peptide, emphasizing the specificity of the response on Q-peptide versus control scrambled peptide. Studies report that bandages removing cytokines such as MCP-1 by electrostatic interactions accelerated wound closure in *db/db* mice.^26^

### Conditioned media from fibroblasts grown on Q-peptide hydrogel attenuate inflammatory cytokine secretion in macrophage-like cells

As wound healing *in vivo* represents a complex process involving interactions between multiple cell types, including immune cells, we sought to study the effect of Q-peptide on one such interaction between fibroblasts and macrophage-like cells (THP-1). When cultivated with culture media conditioned for 1 day with fibroblasts grown on various substrates (TCP, peptide-free hydrogel, scrambled peptide hydrogels, or Q-peptide hydrogels) THP-1 macrophage-like cells (differentiated for 72 h) secreted significantly less IL-6 and exhibited a trend toward lower IL-8 secretion on Q-peptide hydrogels compared to the controls (Figure S2).

### Q-peptide hydrogel attenuates fibrotic cytokine secretion

**Figure 6:**
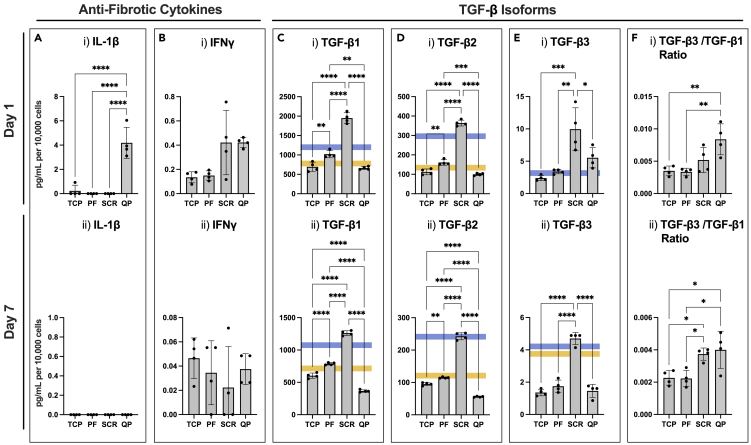
Fibroblasts cultivated on Q-peptide gel exhibit enhanced secretion of anti-fibrotic cytokines (A–F) Cytokine release of anti-fibrotic cytokines (A) IL-1β, (B) IFN-γ, and TGF-β isoforms (C) TGF-β1, (D) TGF-β2, (E) TGF-β3, and (F) TGF-β3/TGF-β1 ratio at (i) 1 day and (ii) 7 days of culture. Surfaces include tissue-culture plastic (TCP), peptide-free hydrogel (PF), scrambled Q-peptide hydrogel (SCR), and Q-peptide hydrogel (QP). Concentration (pg/mL) for IL-1β, and IFN-γ expressed after subtraction of baseline media control and normalized to cell number (10,000 cells). Concentration (pg/mL) for TGF-β1-3 normalized to cell number (10,000 cells). Blue and yellow lines indicate the range of concentrations of TGF-β family cytokines measured in the starting culture media (or just blue line when consistent amongst replicates). Statistical analysis included One-way ANOVA followed by a Tukey’s post hoc test. n = 4. Data presented as mean ± SD ∗ = p < 0.05, ∗∗ = p < 0.01, ∗∗∗ = p < 0.001, ∗∗∗∗ = p < 0.0001.

Next, we sought to study the impact of the Q-peptide hydrogel on fibrosis promoting and attenuating cytokines using ELISA. IL-1β was significantly increased on day 1 when HDFs were cultured on the Q-peptide hydrogel (Figure 6A). This difference was lost by day 7. There was no significant difference in IFN-γ secretion among the groups on either day 1 or day 7 (Figure 6B).

In addition to the above cytokines, an ELISA panel for the three isoforms of TGF-β was also performed. TGF-β1 and TGF-β2, which are implicated in fibrosis pathways, were secreted at a significantly higher concentration in the scrambled peptide hydrogel group on day 1 compared to the other two groups. By day 7, fibroblasts grown on the Q-peptide hydrogel had a significantly lower concentration of TGF-β1 and TGF-β2 compared to the other treatment groups suggesting the Q-peptide hydrogel plays a role in attenuating the concentration of these fibrotic cytokines (Figures 6C and 6D). Of interest, TGF-β3, which is implicated in anti-scarring pathways, was significantly higher in the scrambled peptide hydrogel on day 1 and 7 (Figure 6E). Yet, the ratio of TGF-β3/TGF-β1 was significantly higher on Q-peptide hydrogel compared to TCP and peptide-free hydrogel groups on both days, with a comparable ratio to that of the scrambled peptide (Figure 6F).

### Q-peptide hydrogel enhances fibroblast gene expression profile of wound healing mediators

**Figure 7:**
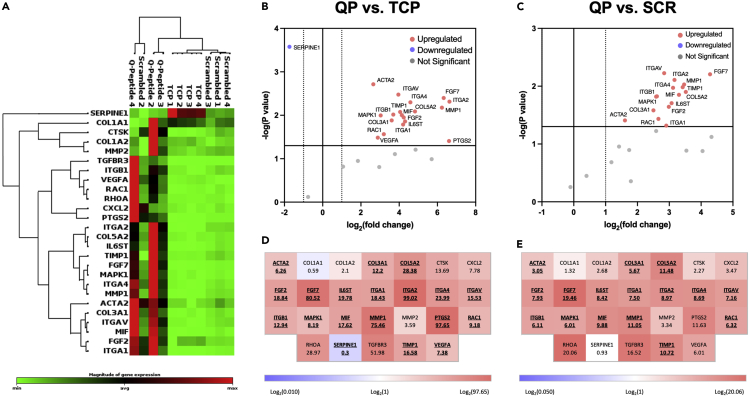
Fibroblasts cultured on Q-peptide hydrogels show enhanced expression profile of wound healing-related genes (A) Clustergram analysis of a wound healing qPCR array comparing the expression profile of fibroblasts grown on TCP to that of Q-and scrambled Q-peptide hydrogels. (B and C) Volcano plots highlighting significantly upregulated and downregulated genes in fibroblasts grown on (B) QP versus TCP and (C) QP versus SCR. (D and E) Wound healing array heatmaps depicting gene symbols and fold changes for (D) QP versus TCP and (E) QP versus SCR. Bold and underlined symbols indicate statistically significant differences. For all plots: n = 3 or 4, p < 0.05 is considered significant.

To further postulate how our *in vitro* observations of fibroblasts cultivated on Q-peptide hydrogel might contribute to enhancing more complex biological processes related to *in vivo* wound healing, we performed a wound healing qPCR array analysis on RNA collected from cells on day 1 of culture. In comparing the expression profile of 26 wound healing-related genes, fibroblasts grown on Q-peptide hydrogels exhibited a clustered and generally upregulated profile distinct from cells grown on TCP and scrambled peptide hydrogels (Figure 7A).

Specific differential expression of wound healing-related genes was assessed on volcano plots and heatmaps (Figures 7B–7E). In particular, 18 of the 26 assayed genes were significantly upregulated in Q-peptide hydrogel cultivated fibroblasts over those grown on TCP (Figure 7B), In contrast, only 3 out of 26 assayed genes were upregulated on scrambled-peptide hydrogel compared to the TCP control (*MMP1* and integrin subunit α2 and β1); whereas collagen genes (*COL1A1, COL1A2*) were downregulated in comparison to the TCP control. A subset of 15 genes that were upregulated on Q-peptide versus TCP (Figures 7B and 7, Table S1) were also significantly upregulated in fibroblasts grown on the Q-peptide hydrogel compared to the scrambled peptide hydrogel (Figures 7C and 7, Table S1).

Consistently upregulated genes in the Q-peptide group, in comparison to both controls (TCP and scrambled) included subunits of important integrin receptors (*ITGA1 ITGA2, ITGA4, ITGAV, ITGB1*) implicated in angiogenesis and migration of keratinocytes during wound healing (Figures 7B and 7C, Table S1).^27^ MAP kinase 1 gene expression, critical for the wound healing process,^28^ was upregulated in the Q-peptide group consistent with upregulation of important integrin subunits. In addition, Q-peptide hydrogel promoted upregulation of growth factors critical in wound healing, over both TCP and scrambled peptide control, specifically fibroblast growth factor (FGF) 2, required for stable vascularization and *FGF7* (aka the keratinocyte growth factor) which is known to promote keratinocyte proliferation.^29^ IL-6 cytokine family signal transducer was also upregulated on Q-peptide hydrogels, consistent with the critical role of IL-6 signaling for timely resolution of wound healing.^30^ Genes for collagens secreted during scarless healing such as *COL3A1*,^31^^,^^32^ and collagens important for stabilizing epidermal/dermal interface as well as enzymes involved in matrix remodeling during wound healing such as *MMP1* and *TIMP1* were uniquely upregulated in dermal fibroblasts cultivated on Q-peptide hydrogel in comparison to both TCP and Scrambled peptide control (Figures 7B and 7C, Table S1). *RAC1*, a GTPase, essential for wound healing that promotes keratinocyte migration and proliferation during wound re-epithelialization was only upregulated on Q-peptide hydrogel in comparison to the two controls. In addition, smooth muscle α−2 actin was upregulated in the Q-peptide hydrogel compared to the controls, consistent with the presence of myofibroblasts required for secretion of extracellular matrix components.

Several additional genes related to wound healing were upregulated in fibroblasts cultivated on Q-peptide compared to the TCP control: *VEGFA*, which is essential in angiogenesis, *PTGS2*, with reported reparative roles in many tissues^33^ and *MIF1* which is upregulated in cutaneous wound healing and considered to promote repair.^34^ Only one gene, *SERPINE1*, was significantly downregulated in Q-peptide fibroblasts (Figure 7B) vs TCP; however, this effect was conserved for cells grown on scrambled Q-peptide gels (Figure S3).

## Discussion

To support the translation of Q-peptide hydrogels to the clinic, it is necessary to validate their direct impact on adult HDFs, which are required for wound healing, but also hold a potential to induce prolonged inflammation, fibrosis and scarring if inappropriately activated by the biomaterial. By studying dermal fibroblasts on Q-peptide and peptide-free hydrogel cast plates, it was possible to assess the impact of the hydrogel directly on these cells.

We observed a time dependent cytokine release that involved secretion of appropriate inflammation and ECM deposition mediators, including key anti-fibrotic and anti-inflammatory cytokines with HDFs cultured on the Q-peptide hydrogel compared to the peptide-free control hydrogels, scrambled peptide hydrogels and TCP. All biomaterials in contact with the body will cause some level of inflammatory response that is often beneficially linked to angiogenesis,^35^ yet its quick resolution supported by the con-current anti-inflammatory cytokine secretion, promises to uniquely support scar-free healthy and rapid wound healing.

Here, we investigated the acute, initial, effects of biomaterials on the HDF within the first day of application because early response of wound bed cells to the applied biomaterial is critical to the final outcome of the healing process. This early enhanced effect can be advantageous for initiating the wound healing response, and the lack of an effect by Day 7 avoids the possible dangers of an extended cytokine release, in the absence of re-applied hydrogel. The “on/off” cytokine release correlating with the hydrogel presence also offers the opportunity to further tailor the response *in vivo* by continued re-application.

Importantly, the results from the microscopy experiments indicated Q-peptide did not induce myofibroblast differentiation or an excessive proliferative response in the dermal fibroblasts. While differentiation and proliferation are necessary for stages of the wound healing process, they can also lead to aberrant scar formation.^19^α-SMA expression is characteristic of myofibroblasts and upregulates HDF contractile activity.^36^ In addition, scar contraction is attributed to α-SMA expressing myofibroblasts.^37^ Lower level of α-SMA on the hydrogel materials is likely because of an optimized substrate stiffness, as TCP has been demonstrated to induce myofibroblast differentiation as a result of its increased stiffness.^38^ Specifically, our previous studies demonstrate that the Q-peptide and peptide-free hydrogel demonstrate G′ or Young’s modulus of 45.37 ± 4.29 Pa,^39^ which is more aligned with the reported elasticity of skin which ranges from 0.1 to 10 kPa,^38^ versus TCP that has been reported to have the Young’s modulus on the order of 1 GPa.^38^^,^^40^ In addition, enhanced collagen IV secretion at early time points on the TCP group is also likely related to the higher stiffness of TCP substrate.

The diverse panel of cytokines is relevant to an acute pro-healing response in dermal fibroblasts. As an early intervention in the wound healing process, Q-peptide could initiate a pronounced signaling response that would be beneficial to wound healing. We were interested in studying the effect of the Q-peptide hydrogel on inflammatory cytokines, ECM-deposition cytokines, and fibrosis promoting cytokines, as all three groups play an important role in skin health and healing. During any healing process, inflammation is necessary but needs to be highly regulated.^41^^,^^42^ Of particular note is TNF-α, a pro-inflammatory cytokine that is integral for sustaining activation of fibroblasts and keratinocytes,^43^ and IL-2 which is an important T-cell growth factor that may impact wound strength and cellular infiltration.^44^ In addition, this cytokine may influence fibroblast metabolism, to promote a positive phenotype for wound healing.^45^

A few of these cytokines are also considered pro-fibrotic in addition to inflammatory, however, a degree of fibrosis and ECM deposition is necessary for proper wound healing^1^^,^^46^ and the ultimate outcome will depend on the ratio of pro-fibrotic and anti-fibrotic cytokines as well as the pro-inflammatory and anti-inflammatory cytokines. IL-6 is necessary for wound closure, as delayed closure was observed in IL-6 knock-out mice, as well as dysregulated α-SMA production.^47^ The downregulation of IL-8 is associated with neonatal scarless wound healing,^48^ and the decrease from Day 1 to Day 7 in Q-peptide hydrogel shows this pathway is not aberrantly activated. Dysregulation and an extended release of IL-8 and IL-6 would be indicative of maintained inflammation and excessive ECM deposition.^49^

Conversely, there were several anti-inflammatory and anti-fibrotic cytokines that were measured in this study. IL-13 is generally considered a master regulator of ECM deposition and, unsurprisingly, is often dysregulated in fibrotic diseases. It is, however, integral for healthy healing in damaged tissues and is considered an immune modulator.^50^^,^^51^ Q-peptide hydrogel has a notable impact; an increased release of IL-13 after 24 h suggesting there is an interaction with these pathways. Similarly, IL-10 is immunosuppressive and downregulates the chronic inflammatory responses through many mechanisms.^51^ It also decreases collagen deposition in dermal fibroblasts possibly by inhibiting matrix metalloproteinases.^52^ We see an important increased release at Day 1 that is not sustained by the Q-peptide hydrogel. In *in vivo* studies and clinical trials, the treatment of GM-CSF was proven to be beneficial in wound healing processes,^53^ as it is involved in signaling re-epithelialization in keratinocytes.^54^ However, a primary function of GM-CSF is also to recruit macrophages and dendritic cells to the wound site, therefore without the recruitment of the immune cells in these culture conditions, the TGF-β pathway, which is responsible for fibrotic regulation, may be inhibited.^55^ Interleukin-5 (IL-5), when overexpressed, results in altered wound healing with prolonged inflammation and ECM deposition.^56^ Similarly, interleukin-4 (IL-4) activates fibroblasts and leads to enhanced ECM deposition,^57^ and IL-1RA is involved in the hypertrophic scar formation after prolonged upregulation or exposure.^58^ Importantly the observed negative impact on fibrosis is temporal, as early expression is needed for normal wound healing to occur.^59^ After seven days these trends are subdued with Q-peptide hydrogel having no significant difference. The P40 subunit of IL-12 is antagonistic to the heterodimer conformation of P70 as it acts competitively for receptors, thus its function is ECM deposition promoting.^60^ Q-peptide appears to have initiated an enhanced response of IL-12 P40 on Day 1.

To study whether the Q-peptide hydrogel has a potential to attenuate fibrosis, IL-1β, IFN-γ, and TGF-β concentrations were measured. IL-1β impacts the deposition of collagen and hyaluronan^61^ and IFN- γ, an anti-fibrotic cytokine, upregulates stromelysin-1^62^ and is involved in signaling pathways to sustain the activation of keratinocytes.^63^^,^^64^ An increase in these cytokines at day 1 on the Q-peptide hydrogel suggests possible mechanisms and pathways that attenuate fibrosis. TGF-β is implicated in myofibroblast differentiation, collagen synthesis and ultimately long-term outcomes of wound healing.^46^^,^^64^^,^^65^ A decrease in fibrosis promoting factors, TGF-β1 and TGF-β2, and an increase in the scar attenuating TGF-β3 in cells cultured on the Q-peptide hydrogel further supports the effect of the Q-peptide hydrogel in reducing fibrosis and promoting a pro-healing environment. Although the ratio of TGF-β3 to TGF-β1 was similar in cells cultivated on Q-peptide and scrambled peptide, scrambled peptide cultivated cells expressed essentially no inflammatory cytokines and their gene expression of wound healing mediators was only slightly different from that on cells cultivated on TCP controls.

Reinforcing these assertions, analyses of gene expression using a wound healing qPCR microarray revealed a distinctly clustered and generally enhanced expressional profile for fibroblasts at day 1 on Q-peptide gels when compared to TCP or scrambled Q-peptide. The upregulated integrin subunits in Q-peptide group may suggest the involvement of the following integrin receptors: α1β1, α2β1, α4β1 and αvβ1, all of which have been implicated in appropriate and effective wound healing response.^66^ The α1β1 integrin mediates VEGF induced angiogenesis and negative feedback regulation of collagen expression. α2β1 has been reported to mediate keratinocyte migration and VEGF-driven angiogenesis and contributes to collagen polymerization by fibroblasts. In the context of wound healing, α4β1 interaction with EMILIN1 may control fibroblast proliferation and TGF-β1 processing and αvβ1 promotes keratinocyte adhesion during wound healing.^66^ Consistent with the well known integrin-MAPK signaling, qPCR also demonstrated upregulation of *MAPK1*.

Growth factors in the FGF family have been extensively studied in wound healing pathways, making the significant upregulation of *FGF2 and FGF7* of particular note. Growth factors encoded by these genes have been recognized for their promotion of keratinocyte re-epithelialization of wounds as well as anti-scarring and anti-fibrotic effects mediated by inhibition of TGF-β2 and α-SMA,^67^ potentially suggesting a contribution from FGF modulation to some of the trends observed in ELISA and immunostaining analyses of these factors. Scarless healing associated collagen 3 was only upregulated in the Q-peptide group, with collagen 5 which promotes dermis/epidermis association. Other upregulated genes such as *RAC1*, *MMP1*, and *PTGS2* may play a more complex role in Q-peptide mediation of both healing and inflammation. *RAC1* has an essential role in wound healing and, further, it promotes keratinocyte migration and proliferation during wound re-epithelialization. Upregulation of *MMP1* is key at day 1 of wound healing but can play a role in chronic non-healing if expression does not decline thereafter,^68^ necessitating the upregulation of its inhibitor *TIMP1*, which was observed on Q-peptide. Expression of *PTGS2*, the gene encoding cyclooxengenase-2 (COX-2), is essential for re-epithelialization and angiogenesis during the early phase of healing but can also be associated with disease-related prolonged inflammation.^69^^,^^70^ When compared with TCP, Q-peptide uniquely upregulated *MIF*, which has been known to either promote fibroblast and keratinocyte migration for improved healing, or to reduce fibroblast migration while increasing inflammatory cytokine release and healing time, depending on the timing and degree of its expression.^34^^,^^71^

### Limitations of the study

Although more work is needed to definitively determine the pathways responsible for the accelerated healing observed within *in vivo* models,^9^^,^^10^ it is clear from our work with dermal fibroblasts that the response is independent yet interconnected. As a whole, qPCR analysis points at significantly enhanced *in vivo* wound healing potential for fibroblasts grown on Q-peptide gels, however further studies are required to assess the mechanistic implications of highlighted genes both *in vitro* and in a more complex *in vivo* wound context. Lack of extended cytokine release by Day 7, also points to the importance of the material presence in achieving a sustained response, as most of the solvent-cast material was released by design within the first 24 h thus the cytokine response was not prolonged to seven days. It would also be important to confirm the described findings in a model consisting of both an epidermis and a dermis.

### Conclusion

The cytokine and microscopy analysis provides insight into the complex and interconnected signaling involved in wound healing. The apparent difference in almost all cytokines, particularly at Day 1, highlights the profound impact of Q-peptide conjugated hydrogel on the adult HDF secretome. This impact includes a temporally appropriate response promoting ECM deposition and both anti-inflammatory and anti-fibrotic cytokines. In addition, we have identified key cytokines involved in sustaining communication with both keratinocytes and immune cells and 15 genes implicated in wound healing that were uniquely upregulated on the Q-peptide hydrogel. All together these results suggest a multifaceted and complex pro-healing activation of normal adult human dermal fibroblasts with the Q-peptide hydrogel.

## STAR★Methods

### Key resources table

**Table:** 

REAGENT or RESOURCE	SOURCE	IDENTIFIER
**Antibodies**		

Ki-67 Antibody	Cell Signaling Technology	Cat#12075S; RRID: AB_2728830
4′,6-diamidino-2-phenylindole (DAPI)	Invitrogen	Cat#D1306; RRID: AB_2629482
Vimentin Antibody	Abcam	Cat#ab8978; RRID: AB_306907
SMA Antibody	Abcam	Cat#ab5964; RRID: AB_2223021
Collagen IV Antibody	Abcam	Cat#ab6586; RRID: AB_305584
Alexa Flour 488	Abcam	Cat#ab150113; RRID: AB_2576208
Alexa Flour 647	Invitrogen	Cat#A21245; RRID: AB_2535813

**Chemicals, peptides, and recombinant proteins**		

Q-peptide	Genscript	Custom Synthesis
Chitosan	Heppe Medical Chitosan	Cat#4401
Scrambled Peptide	Biomatik	Custom Synthesis
DPBS	Sigma-Aldrich	Cat#D1048-500 ML
EDC	ThermoFisher Scientific	Cat#22980
S-NHS	ThermoFIsher Scientific	Cat#24510
Acetic Acid	Caledon	Cat#1000-1-29
Type 1 Rat Tail Collagen	Corning	Cat#354236
FITC tagged Q-peptide	GenScript	Custom Synthesis
phorbol 12-myristate-13-acetate (PMA)	Sigma-Aldrich	Cat#: P8139-5 MG
PBS	Sigma-Aldrich	Cat#D8537-500 ML
Carboxyflourescein Diacetate, Succinimidyl Ester (CFDA-SE)	Invitrogen	Cat#C1157
Propidinium Iodide (PI)	Invitrogen	Cat#P3566
Paraformaldehyde	Thermo Fisher Scientific	Cat#28908
Qiazol	Qiagen	Cat#79306
2x RT^2^ SYBR® Green ROX qPCR Mastermix	Qiagen	Cat# 330522

**Critical commercial assays**		

Mycoplasma Contamination Kit	Lonza	Cat#LT07-118
Human IL-6 ELISA Kit	Sigma-Aldrich	Cat#RAB0306
Human IL-8 ELISA Kit	Sigma-Aldrich	Cat#RAB0319
The Human Cytokine Array Pro-inflammatory Focused 15-plex HDF15	Eve Technologies	N/A
RT^2^ First Strand Kit	Qiagen	Cat#330401
RT^2^ Profiler™ PCR Array, Human Wound Healing	Qiagen	Cat# 330231, PAHS-121ZE-4

**Deposited data**		

Raw Data and Analysis	This Paper	N/A

**Experimental models: Cell lines**		

THP-1 Cells	ATCC	Cat#TIB-202
HDF Cells	Angio-Proteomie	Cat#cAP-0008-ad

**Software and algorithms**		

ImageJ	Schneider et al.^72^	https://imagej.nih.gov/ij/
Prism	Mavrevski et al.^73^	https://www.graphpad.com/scientific-software/prism/

**Other**		

RPMI	Wisent	Cat#350-007-CL
FBS (for RPMI Medium)	ScienCell	Cat#0010
DMEM	GIBCO	Cat#11960-044
Penicillin/Streptomycin	GIBCO	Cat#15140-122
FBS (for DMEM Medium)	GIBCO	Cat#12483-020

### Resource availability

#### Lead contact

Further information and requests for reagents may be directed to, and will be fulfilled by the lead contact, Dr. Milica Radisic (m.radisic@utoronto.ca).

#### Materials availability

This study did not generate new unique reagents.

### Experimental model and subject details

#### Cell lines

Tohoku Hospital Pediatrics-1 (THP-1, male, 1 year old) cells were cultured in RPMI ([RPMI]; Wisent, Montreal, QC, Canada; Cat#: 350-007-CL) containing 10% fetal bovine serum (ScienCell, Carlsbad, CA, USA, Cat#: 0010) and maintained at 37°C in a 5% CO2 humidified incubator. Cells were maintained in T175 Nunc EasYFlask Cell Culture Flasks (ThermoFisher, Waltham, MA, USA, Cat#: 159910) at a cell density between 1x10^5^ cells/ml and 1x10^6^ cells/ml (passages 10–12 were used). Source cell vials were monitored and subsequently tested negative for the presence of mycoplasma contamination (Lonza, Gampel, GB, Switzerland, Cat#: LT07-118). STR profiling for cell line authentication (GenePrint 10 System, Promega) was performed by The Center for Applied Genomics (TCAG), The Hospital for Sick Children, Toronto, Canada (Table S2). DNA was isolated from cells using the PureLink Genomic DNA Mini Kit (Invitrogen, Cat #K182001) according to the manufacturers protocol, quantified via Nanodrop, and diluted to a concentration of 30 ng/μL for profiling.

#### Primary cultures

##### Normal adult human dermal fibroblasts

Human adult fibroblasts (HDF, female, 45 years old) were purchased from (Angio-Proteomie Cat# cAP-0008-ad). Cell cultivation was performed under a University of Toronto CL2 Biosafety Permit 220-R01-2according to standard operating procedures. Briefly, HDF were grown Dulbecco's Modified Eagle's Medium (DMEM) (GIBCO Cat#11960-044) supplemented with 1% penicillicin/streptomycin (Gibco Cat # 15140-122) and 10% fetal bovine serum (FBS) (Gibco Cat #12483-020) and maintained at 37°C in a 5% CO2 humidified incubator. Cells were passaged at 80–100% confluency, and passage number seven to thirteen was used for experiments. HDF cells were seeded at 2.6x10^3^ cells/cm^2^ for experiments. STR profiling was performed as described above (Table S2).

### Method details

#### Peptide modified chitosan conjugation

The Q-Peptide (QHREDGS; Genscript) was conjugated to chitosan using 1-ethyl-3-(3-dimethylaminopropyl) carbodiimide (EDC) chemistry.^72^ In this reaction the primary carboxyl groups of QHREDGS peptide react with Sulfo-NHS when EDC is present resulting in the formation of amide bonds on the chitosan backbone. To make a thermo-sensitive hydrogel, the peptide-conjugated chitosan is purified by dialysis and combined with collagen.

Prior to conjugation, the chitosan (Heppe Medical Chitosan) was dissolved in 0.9% normal saline at 20 mg/mL and Q-Peptide was dissolved in PBS at 10 mg/mL. EDC (ThermoFisher Scientific Cat#22980) N-hydroxysulfosuccinimide (S-NHS; ThermoFisher Scientific Cat#24510) were dissolved, separately, in PBS and mixed with the chitosan and QHREDGS peptide to achieve a final concentration of 5 mg/mL and 3 mg/mL respectively. The solution was vortexed for 3h, and diluted 4x in PBS before being dialyzed against distilled, deionized water for 24h. The solution was sterile filtered and lyophilized for 48h. Lyophilized material was stored at −20°C until further use. The same procedures were repeated to create scrambled Q-Peptide (DGQESHR, Biomatik) modified chitosan.

#### Solvent casting of chitosan-collagen films

For *in vitro* experiments, chitosan-collagen films were solvent cast in cell culture well plates. Chitosan (peptide-free, with conjugated Q-Peptide, or with scrambled Q-Peptide) was dissolved in 0.5N acetic acid at 2 mg/mL and mixed with 2 mg/mL type 1 collagen. Non-adherent plates were used for casting. 24-well plates and the 6-well plates were coated with 250 μL and 750 μL per well, respectively. The coating solution was allowed to completely evaporate in a biosafety hood, leaving behind a film of chitosan-collagen with and without peptide conjugation. Prior to use, the films were washed 3x with PBS.

#### Material degradation characterization

Fluorescein isothiocyanate (FITC) tagged Q-Peptide was conjugated to chitosan and solvent cast to 24-well polystyrene plates as previously described.^13^ Briefly, chitosan (with or without conjugated Q-Peptide or scrambled Q-Peptide) was dissolved in 0.5 N acetic acid at 2 mg/mL and mixed with 2 mg/mL type 1 collagen. Non-adherent 6-well plates were coated with 750 μL of material per well. The coating solution was allowed to fully evaporate in a biosafety hood, leaving behind a chitosan−collagen film. Prior to use, the films were washed three times with PBS.There was constant consideration for the light sensitive nature of the material, so the plates were carefully wrapped in aluminum foil during storage and the experiments. The cast plates were washed three times with DPBS and fluorescence was measured with a spectrophotometer (BioTek Cytation5) with an excitation at 490 nm and emission at 520 nm. After 24 h, the supernatant was collected, and the fluorescence of the material with and without cells was measured in DPBS. The percentage of released material was calculated (Figure S1).

$$Material\;Released\;\left(\%\right)=100-\left(\frac{\left(Fluorescence\;on\;Day\;1\right)}{Fluorescence\;on\;Day\;0}\times 100\right)$$

#### Microscopy

After 24 h, HDF cells were sampled for Live-Dead staining. Cells were incubated with Carboxyfluorescein Diacetate, Succinimidyl Ester (CFDA-SE, Invitrogen Cat #C1157) and propidinium iodide (PI; Invitrogen Cat# P3566) for 10 min prior to imaging according to the manufacturer’s instructions (Invitrogen). Cells were imaged within 20 min to avoid detachment.

HDF cells were fixed with 4% paraformaldehyde. Cells were stained with the proliferation marker, Ki-67 (Cell Signaling Technology Cat# 12075S; ½00 dilution) and counterstained with 4′,6-diamidino-2-phenylindole (DAPI; Invitrogen Cat# D1306; 1:1000). Fibroblasts were stained with the following primary antibodies: cytoskeletal marker mouse-*anti*-Vimentin (Abcam Cat# ab8978; ½00 dilution), rabbit-*anti*-smooth muscle actin (SMA; Abcam Cat# ab5964; ½00 dilution), collagen IV (Abcam Cat# ab6586; ½00 dilution), and DAPI. A secondary fluorophore conjugated antibody of Alexa Fluor 488 (Abcam Cat# ab150113; 1/100 dilution) or Alexa Fluor 647 (Abcam Cat# ab21245; 1/100 dilution) was used for visualization of the structures. The cells were imaged using an Olympus CKX41 inverted microscope and CellSens software (Olympus Corporation).

Images were analyzed using ImageJ. Where cells were counted, images were reduced to an 8-bit binary image and the Cell counter tool was used to count the cells. Other tools such as watershed were used to separate close cells. For αSMA and Ki67 positive cells, the following equation was used to determine the percentage of positive cells.

$$Positive\;Cells\;\left(\%\right)=\frac{Positive\;Cells}{Positive\;Cells+Negative\;Cells}\times 100$$

For the Vimentin analysis where the Aspect Ratio (AR) was compared, the Cell Counter tool expanded to include an automatic calculation AR in addition to cell number for Day 1 and Day 7. Calculation for AR is as shown below.

$$Aspect\;Ratio\;\left(AR\right)=\frac{Largest\;length\;of\;Cell}{Smallest\;Length}$$

#### Cytokine preparation and analysis

The cytokine analysis was performed using ELISA or microspheres by Eve Technologies (Calgary, AB). The Human Cytokine Array Pro-inflammatory Focused 15-plex HDF15 (GM-CSF, IFNγ, IL-1β, IL-1ra, IL-2, IL-4, IL-5, IL-6, IL-8, IL-10, IL-12(p40), IL-13, MCP-1, and TNFα) and the TGF-β 3-plex (TGF-β 1, 2, and 3). Background media concentration was subtracted from each cytokine concentration and normalised to the cell number. Cell numbers at Day 1 and Day 7, for the normalization of cytokine data, was determined using ImageJ analysis of Vimentin-stained cells. The cell counter tool was used to distinguish and count nuclei in the images with DAPI only.

#### qPCR

Total RNA was isolated from HDF cells cultured on TCP or chitosan-collagen gels conjugated with Q-Peptide or scrambled Q-Peptide on day 1 of culture using Qiazol (Qiagen, cat#79306) according to the manufacturers protocol. Briefly, cells were seeded in a 6-well plate in quadruplicates and 1 mL of Qiazol was applied to each well. Chloroform was used for phase separation, followed by isopropanol precipitation and two washes with ethanol.

Isolated total RNA was quantified via NanoDrop. 100 ng of total RNA from each sample was taken for cDNA preparation using the RT^2^ First Strand Kit (Qiagen cat# 330401) according to the manufacturers protocol. 102 μL of cDNA was mixed with 650 μL of 2x RT^2^ SYBR Green ROX qPCR Mastermix (Qiagen cat# 330522) and 548 μL RNase free water. 10 μL of cDNA/mastermix was added to each well of the RT^2^ Profiler PCR Array, Human Wound Healing (Qiagen cat# 330231, PAHS-121ZE-4). qPCR was performed on a Bio-Rad CFX384 Real-Time PCR system starting with 10minat 95°C followed by 40 cycles of 15sat 95°C, 60sat 60°C, and fluorescence detection. Data analysis was performed using the GeneGlobe online analysis platform (Qiagen) using the ΔΔC_t_ method. All genes were normalized to GAPDH with the TCP group serving as a control for Q-Peptide and scrambled Q-Peptide groups. As a component of our hydrogel (chitosan) tends to bind strongly to nucleic acids due to its positive charge, stringent quality control was used to eliminate any possible genomic DNA contamination. For quality control purposes, all genes that exceeded the C_t_ value of the panel’s built-in genomic DNA control for any given sample (C_t_ values ranging from 30.8 to 40 depending on sample) were omitted for all samples such that only genes with relatively low C_t_ values were used for downstream analyses. This resulted in 26 genes that could be reliably quantified, see Data S1.

#### THP-1 cell culture experiments

To conduct stimulation experiments, THP-1 cells, between passages 8–9, were seeded at a density of 4x10^5^ cells/ml in 24-well plates (Corning Life Sciences, Corning, NY, USA) and differentiation to THP-1 macrophage-like cells conducted using established protocols. Briefly, THP-1 cells were incubated for 72 h with 100 nM phorbol 12-myristate-13-acetate ([PMA]; Sigma-Aldrich, St Louis, MO, USA). Subsequent THP-1 macrophage-like cells were washed twice in warm sterile Dulbecco’s−/−PBS (Sigma-Aldrich, St Louis, MO, USA, D8537-500 ML, Cat#: P8139-5 MG) and rested in complete RMPI media for 24 h. Conditioned media from fibroblast cultures collected on day 1 were subsequently strained through 70 μm cell strainers and diluted 50% v/v with RMPI and incubated with the THP-1 macrophage-like cells for 24 h to gauge inflammatory response. Media was subsequently collected and spun at 1,000 x g, 4°, for 5 min to deplete any potential cellular carryover. IL-6 and IL-8 ELISA was carried out using Sigma-Aldrich kits (Cat#RAB0306 and RAB0319). The delta concentrations of cytokines post-treatment versus pre-treatment (in the 50:50 media initially applied to THP-1 cells) were calculated and plotted for comparison.

### Quantification and statstical analysis

All results are presented as mean ±SD Statistical analysis was performed using GraphPad Prism 6. Student’s *t*test, or one-way or two-way ANOVA, followed by a Tukey post hoc test for pairwise comparison was used to calculate the differences between experimental groups where indicated. A value of p< 0.05 was considered statistically significant. ∗ = p < 0.05, ∗∗ = p < 0.01, ∗∗∗ = p<0.001, ∗∗∗∗ = p<0.0001.

### Additional resources

None.

## Data Availability

• Relevant data reported in this paper will be shared by the lead contact upon request. • This paper does not report original code. • Any additional information required to reanalyze the data reported in this paper is available from the lead contact upon request.

## Supplemental information

Document S1. Figures S1–S3 and Tables S1 and S2

Data S1. Raw data and analyses of wound healing qPCR microarray, related to Figure 7
