# Supplementary material for: Angiopoietin-1 derived peptide hydrogel promotes molecular hallmarks of regeneration and wound healing in dermal fibroblasts
Source: iScience. 2023 Jan 14;26(2):105984. doi: 10.1016/j.isci.2023.105984 (PMC9932487; doi:10.1016/j.isci.2023.105984)
Supplement: Document S1. Figures S1–S3 and Tables S1 and S2 [file mmc1.pdf]

iScience, Volume *26*

Supplemental information

Angiopoietin-1 derived peptide hydrogel promotes molecular hallmarks of regeneration and wound healing in dermal fibroblasts

Katrina Vizely, Karl T. Wagner, Serena Mandla, Dakota Gustafson, Jason E. Fish, and Milica Radisic

Supplemental Information

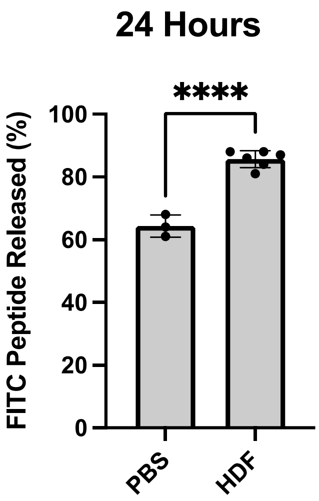

Supplemental Figure 1: Comparison of the release of a FITC-Q-Peptide material over 24 hours, Related to Figure 2. The FITC-peptide was conjugated to chitosan and solvent casted with a neutralized collagen onto tissue culture plates. To measure the amount of material, the fluorescence of the material was measures with an excitation at 490nm and emission at 520nm using a spectrophotometer. Statistical Analysis included a t-test, where n = 3-6 and data are presented as mean ± SD. t-test, **** = $p<0.0001$.

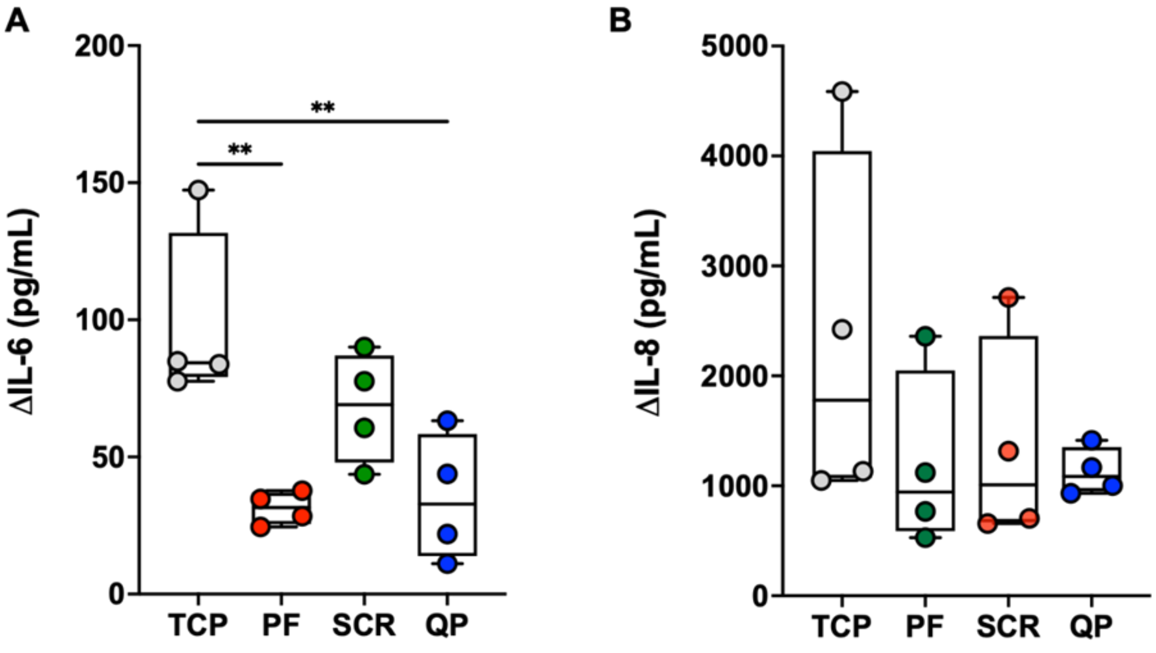

Supplemental Figure 2. Factors secreted by fibroblasts cultivated on Q-peptide hydrogel attenuate inflammatory cytokine secretion by macrophage-like cells, Related to Figure 4. Inflammatory cytokine secretion by THP-1 macrophage-like cells cultivated in culture media conditioned by fibroblasts grown on various substrates (50% THP-1 culture media, 50% fibroblast conditioned media) after 24hr in culture. Values represent the difference between IL-6 and IL-8 values in the initially applied culture media to that measured at the end of cultivation. TCP- tissue culture plastics, PF- peptide free hydrogel, SCR-scrambled peptide hydrogel QP- Q-peptide hydrogel. n = 4, data are presented as box plots extending between the 25th and 75th percentiles of each dataset. Whiskers denote minimum and maximum values, line in middle of box denotes mean. One-way ANOVA, ** = $p<0.01$

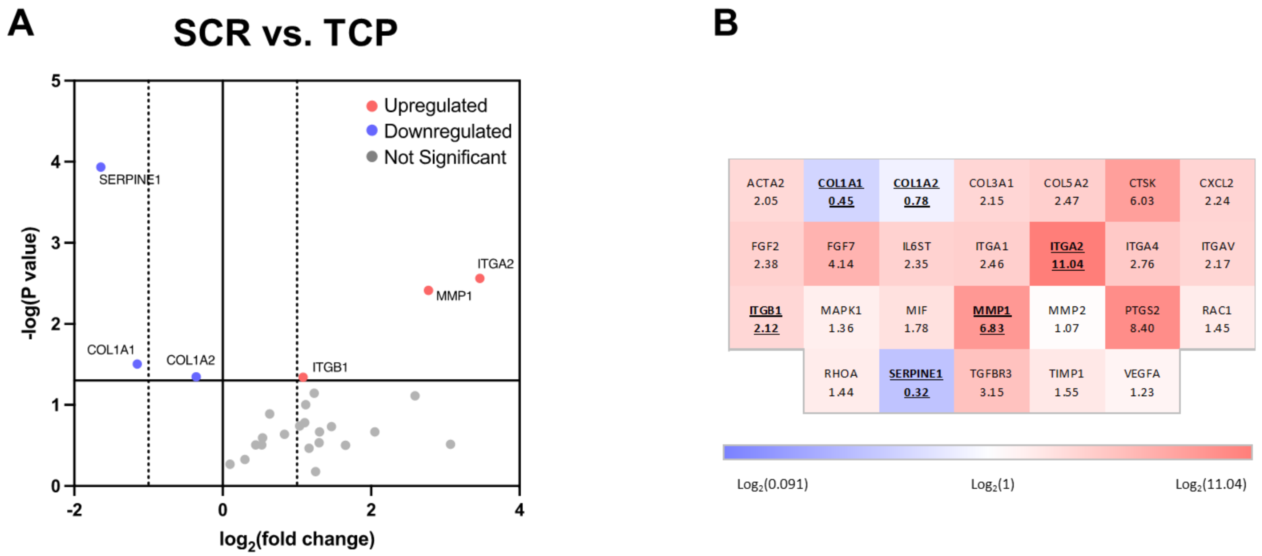

Supplemental Figure 3. Differential gene expression between fibroblasts cultivated on scrambled peptide hydrogel and tissue culture plastics, Related to Figure 7. (A) Volcano plot highlighting significantly upregulated and downregulated genes in fibroblasts grown on SCR vs. TCP. (B) Wound healing array heatmap depicting gene symbols and fold changes for SCR vs. TCP. Bold and underlined symbols indicate statistically significant differences. For all plots: n=3 or 4, P<0.05 is considered significant.

Supplemental Table 1. Gene expression of important wound healing mediators, Related to Figure 7. The shaded area indicates genes at a significantly higher level of expression in human dermal fibroblast on Q-peptide hydrogel compared to the tissue culture plastic. The red box indicates significantly higher gene expression in human dermal fibroblasts cultivated on Q-peptide hydrogel compared to the scrambled peptide hydrogel. The blue box indicates a significantly lower gene expression in human dermal fibroblast on Q-peptide hydrogel compared to the tissue culture plastic.

Gene Symbol	Gene name	Role in wound healing
**ITGA1**	integrin subunit α1	This gene encodes the α1 subunit of integrin receptors. This protein heterodimerizes with the β1 subunit to form a cell-surface receptor for collagen and laminin. The heterodimeric receptor is involved in cell-cell adhesion and may play a role in inflammation and fibrosis. The α1 subunit contains an inserted (I) von Willebrand factor type I domain which is thought to be involved in collagen binding[1]. For example, the α1β1 integrin mediates VEGF induced angiogenesis and negative feedback regulation of collagen expression. α1 null mice exhibit increased expression of collagen during granulation tissue formation[2].
**ITGA2**	integrin α2	This gene encodes the α subunit of a transmembrane receptor for collagens and related proteins. For example, α2β1 mediates keratinocyte migration and VEGF-driven angiogenesis and contribute to collagen polymerization by fibroblasts[1,2].
**ITGA4**	integrin subunit α4	The gene encodes a member of the integrin α chain family of proteins. The encoded preproprotein is proteolytically processed to generate light and heavy chains that comprise the α4 subunit. This subunit associates with a β1 or β7 subunit to form an integrin that may play a role in cell motility and migration. In the context of wound healing, α4β1 interaction with EMILIN1 may control fibroblast proliferation and TGF-β1 processing. α4β7 promotes leukocyte extravasation[1,2].
**ITGAV**	integrin subunit αV	The product of this gene belongs to the integrin α chain family. The encoded preproprotein is proteolytically processed to generate light and heavy chains that comprise the αV subunit. This subunit associates with β1, β3, β5, β6 and β8 subunits. αvβ1 promotes keratinocyte adhesion during wound healing. αVβ3 is essential for neoangiogenesis and pericyte retention during wound haling. αvβ5 may participate in myofibroblast transformation. αvβ6 regulates inflammation and keratinocyte proliferation, as well as basement membrane and granulation tissue remodelling. αvβ8 may contribute to TGF-β activation[1,2].
**ITGB1**	integrin subunit β1	This gene encodes β subunit of integrins which have multiple roles in wound healing. For example, the α1β1 integrin mediates VEGF induced angiogenesis and negative feedback regulation of collagen expression. α1 null mice exhibit increased expression of collagen during granulation tissue formation. α2β1 mediates keratinocyte migration and

		VEGF-driven angiogenesis and contribute to collagen polymerization by fibroblasts[1,2].
**MAPK1**	mitogen-activated protein kinase 1	This gene encodes a member of the MAP kinase family. MAP kinases, also known as extracellular signal-regulated kinases (ERKs), act as an integration point for multiple biochemical signals, and are involved in a wide variety of cellular processes such as proliferation, differentiation, transcription regulation and development. MAP kinase activation is generally considered to promote wound healing[1,3].
**FGF2**	fibroblast growth factor 2	The protein encoded by this gene is a member of the fibroblast growth factor (FGF) family. It promotes angiogenesis and wound healing[1].
**FGF7**	fibroblast growth factor 7	This protein is a potent epithelial cell-specific growth factor, whose mitogenic activity is predominantly exhibited in keratinocytes but not in fibroblasts and endothelial cells. It is also known as keratinocyte growth factor[1].
**IL6ST**	interleukin 6 cytokine family signal transducer	The protein encoded by this gene is a signal transducer shared by many cytokines, including interleukin 6 (IL-6), which is associated with inflammation. IL-6 signaling is required for timely resolution of wound healing[1].
**COL3A1**	collagen type III α 1 chain	Encodes the pro-α1 chains of type III collagen, a fibrillar collagen that provides skin flexibility, softness and tensibility frequently in association with type I collagen. Collagen III to collagen I ratio is increased in normal skin and fetal scarless wound compared to the scar tissue and keloids, where collagen I is more abundant[1,4,5].
**COL5A2**	collagen type V α 2 chain	This gene encodes an α-chain for type V fibrillar collagen, one of the low abundance fibrillar collagens. Type V collagen is found in tissues containing type I collagen and appears to regulate the assembly of heterotypic fibers composed of both type I and type V collagen. Bridges and stabilizes the epidermal–dermal interface and contributes to epidermis differentiation[1,5].
**MMP1**	matrix metallopeptidase 1	This gene encodes a member of the peptidase M10 family of matrix metalloproteinases (MMPs). This secreted protease breaks down the interstitial collagens, including types I, II, and III. It is critical for basement membrane remodelling during wound healing and promotes re-epithelialization[1,5].
**TIMP1**	TIMP metallopeptidase inhibitor 1	The proteins encoded by TIMP gene family are natural inhibitors of the matrix metalloproteinases (MMPs), a group of peptidases involved in degradation of the extracellular matrix. In addition to its inhibitory role against most of the known MMPs, the encoded protein is able to promote cell proliferation in a wide range of cell types, and may also have an anti-apoptotic function. Transcription of this gene is highly inducible in response to many cytokines and hormones. TIMP1 inhibits MMP1, thus regulating matrix remodelling during wound healing[1,5].
**RAC1**	Rac family small GTPase 1	The protein encoded by this gene is a GTPase which belongs to the RAS superfamily of small GTP-binding proteins. Members of this superfamily appear to regulate a diverse array of cellular events, including the control of cell growth, cytoskeletal reorganization, and the activation of protein kinases. It has an essential role wound healing and,

		further, it promotes keratinocyte migration and proliferation during wound re-epithelialization[1,6].
**ACTA2**	actin α 2, smooth muscle	This gene encodes one of six different actin proteins. It marks myofibroblast activation required for ECM deposition and wound contraction. Excess activation could lead to scarring[1].
**VEGFA**	vascular endothelial growth factor A	This gene is a member of the PDGF/VEGF growth factor family. VEGFA induces proliferation and migration of vascular endothelial cells, and is essential for angiogenesis[1].
**PTGS2**	prostaglandin-endoperoxide synthase 2	Prostaglandin-endoperoxide synthase (PTGS), also known as cyclooxygenase, is the key enzyme in prostaglandin biosynthesis, and acts both as a dioxygenase and as a peroxidase. It is found to promote regeneration of many tissues including skin[1,7].
**MIF**	macrophage migration inhibitory factor	This gene encodes a lymphokine involved in cell-mediated immunity, immunoregulation, and inflammation. It plays a role in the regulation of macrophage function in host defense through the suppression of anti-inflammatory effects of glucocorticoids. It is upregulated during cutaneous wound healing and considered to promote repair[1,8].
**COL1A1**	collagen type I α1 chain	Encodes the pro-α1 chains of type I collagen whose triple helix comprises two α1 chains and one α2 chain. Type I is a fibril-forming collagen found in dermis and contributes to maintaining skin structure and tissue integrity. Excess could lead to fibrosis[1,5].
**COL1A2**	collagen type I α 2 chain	Encodes pro-α2 chain of type I collagen whose triple helix comprises two α1 chains and one α2 chain. Type I is a fibril-forming collagen found in dermis and contributes to maintaining skin structure and tissue integrity. Excess could lead to fibrosis[1,5].
**CTSK**	cathepsin K	The protein encoded by this gene is a lysosomal cysteine proteinase and a member of the peptidase C1 protein family, is predominantly expressed in osteoclasts. It exhibits collagenolytic activity and it is more highly expressed in dermal fibroblasts from scars and keloids, whereas normal skin exhibits only low expression levels[1,9].
**CXCL2**	C-X-C motif chemokine ligand 2	This antimicrobial gene is part of a chemokine superfamily that encodes secreted proteins involved in immunoregulatory and inflammatory processes. This chemokine, a member of the CXC subfamily, is expressed at sites of inflammation. Recruits an initial wave of inflammatory cells, such as neutrophils and macrophages, to the wound and promotes angiogenesis. Prolonged expression may impair wound healing[1,10].
**MMP2**	matrix metallopeptidase 2	The protein encoded by this gene is a gelatinase A, type IV collagenase, that contains three fibronectin type II repeats in its catalytic site that allow binding of denatured type IV and V collagen and elastin. Unlike most MMP family members, activation of this protein can occur on the cell membrane. It is important for removal of denatured collagen in wound healing and it can expose a cryptic epitope within collagen IV that promotes angiogenesis[1,5].

**RHOA**	ras homolog family member A	This gene encodes a member of the Rho family of small GTPases, which cycle between inactive GDP-bound and active GTP-bound states and function as molecular switches in signal transduction cascades. Rho proteins promote reorganization of the actin cytoskeleton and regulate cell shape, attachment, and motility. It is required for directed keratinocyte migration[1,11].
**SERPINE1**	serpin family E member 1	This gene encodes a member of the serine proteinase inhibitor (serpin) superfamily. This member is the principal inhibitor of tissue plasminogen activator (tPA) and urokinase (uPA), and hence is an inhibitor of fibrinolysis. Regulator of stromal remodelling in the wound environment. Deficient or elevated levels of SERPINE1 are causative factors in healing anomalies including impaired wound resolution[1,12].
**TGFBR3**	transforming growth factor beta receptor 3	This locus encodes the transforming growth factor TGF-β type III receptor. The encoded receptor is a membrane proteoglycan that often functions as a co-receptor with other TGF-beta receptor superfamily members. The increasing ratio of TGF–β3 signaling is thought to promote scarless wound healing and it is required for healing of excisional wounds[1,13–17].

Supplemental Table 2: Results of STR profiling for cell line authentication, Related to Figure 1 - 7. Profiling was performed using the GenePrint® 10 System (Promega).

**Marker**	**hDF** (Angio-Proteomie cAP-0008-ad)		**THP-1** (ATCC TIB-202)	
	**Allele 1**	**Allele 2**	**Allele 1**	**Allele 2**
AMEL	X	X	X	Y
CSF1PO	12	12	11	13
D13S317	9	12	13	13
D16S539	12	13	11	12
D21S11	30	34.2	30	31.2
D5S818	12	12	11	12
D7S820	8	11	10	10

TH01	7	9	8	9.3
TPOX	10	11	8	11
vWA	15	20	16	16

Supplemental Information References

1. O'Leary, N.A., Wright, M.W., Brister, J.R., Ciufo, S., Haddad, D., McVeigh, R., Rajput, B., Robbertse, B., Smith-White, B., Ako-Adjei, D., et al. (2016). Reference sequence (RefSeq) database at NCBI: current status, taxonomic expansion, and functional annotation. Nucleic Acids Res. *44*, D733-45. 10.1093/nar/gkv1189.
2. Koivisto, L., Heino, J., Häkkinen, L., and Larjava, H. (2014). Integrins in Wound Healing. Adv. wound care *3*, 762–783. 10.1089/wound.2013.0436.
3. Lee, S., Kim, M.S., Jung, S.-J., Kim, D., Park, H.J., and Cho, D. (2018). ERK activating peptide, AES16-2M promotes wound healing through accelerating migration of keratinocytes. Sci. Rep. *8*, 14398. 10.1038/s41598-018-32851-y.
4. Karppinen, S.M., Heljasvaara, R., Gullberg, D., Tasanen, K., and Pihlajaniemi, T. (2019). Toward understanding scarless skin wound healing and pathological scarring [version 1; peer review: 2 approved]. F1000Research *8*, 1–11. 10.12688/f1000research.18293.1.
5. Xue, M., and Jackson, C.J. (2015). Extracellular Matrix Reorganization During Wound Healing and Its Impact on Abnormal Scarring. Adv. wound care *4*, 119–136. 10.1089/wound.2013.0485.
6. DiPersio, C.M. (2007). Double Duty for Rac1 in Epidermal Wound Healing. Sci. STKE *2007*, pe33–pe33. 10.1126/stke.3912007pe33.
7. Cheng, H., Huang, H., Guo, Z., Chang, Y., and Li, Z. (2021). Role of prostaglandin E2 in tissue repair and regeneration. Theranostics *11*, 8836–8854. 10.7150/thno.63396.
8. Farr, L., Ghosh, S., and Moonah, S. (2020). Role of MIF Cytokine/CD74 Receptor Pathway in Protecting Against Injury and Promoting Repair. Front. Immunol. *11*, 1273. 10.3389/fimmu.2020.01273.
9. Rünger, T.M., Quintanilla-Dieck, M.J., and Bhawan, J. (2007). Role of Cathepsin K in the Turnover of the Dermal Extracellular Matrix during Scar Formation. J. Invest. Dermatol. *127*, 293–297. https://doi.org/10.1038/sj.jid.5700535.
10. Ridiandries, A., Tan, J.T.M., and Bursill, C.A. (2018). The Role of Chemokines in Wound Healing. Int. J. Mol. Sci. *19*. 10.3390/ijms19103217.
11. Jackson, B., Peyrollier, K., Pedersen, E., Basse, A., Karlsson, R., Wang, Z., Lefever, T., Ochsenbein, A.M., Schmidt, G., Aktories, K., et al. (2011). RhoA is dispensable for skin development, but crucial for contraction and directed migration of keratinocytes. Mol. Biol. Cell *22*, 593–605. 10.1091/mbc.e09-10-0859.
12. Simone, T.M., Higgins, C.E., Czekay, R.-P., Law, B.K., Higgins, S.P., Archambeault, J., Kutz, S.M., and Higgins, P.J. (2014). SERPINE1: A Molecular Switch in the Proliferation-Migration Dichotomy in Wound-"Activated" Keratinocytes. Adv. wound care *3*, 281–290. 10.1089/wound.2013.0512.

13. Valluru, M., Staton, C., Reed, M., and Brown, N. (2011). Transforming Growth Factor-β and Endoglin Signaling Orchestrate Wound Healing. Front. Physiol. *2*. 10.3389/fphys.2011.00089.
14. Le, M., Naridze, R., Morrison, J., Biggs, L.C., Rhea, L., Schutte, B.C., Kaartinen, V., and Dunnwald, M. (2012). Transforming Growth Factor Beta 3 Is Required for Excisional Wound Repair In Vivo. PLoS One *7*, 1–10. 10.1371/journal.pone.0048040.
15. Occleston, N.L., O'Kane, S., Laverty, H.G., Cooper, M., Fairlamb, D., Mason, T., Bush, J.A., and Ferguson, M.W.J. (2011). Discovery and development of avotermin (recombinant human transforming growth factor beta 3): a new class of prophylactic therapeutic for the improvement of scarring. Wound repair Regen. Off. Publ. Wound Heal. Soc. [and] Eur. Tissue Repair Soc. *19 Suppl 1*, s38-48. 10.1111/j.1524-475X.2011.00711.x.
16. Larson, B.J., Longaker, M.T., and Lorenz, H.P. (2010). Scarless fetal wound healing: a basic science review. Plast. Reconstr. Surg. *126*, 1172–1180. 10.1097/PRS.0b013e3181eae781.
17. Penn, J.W., Grobbelaar, A.O., and Rolfe, K.J. (2012). The role of the TGF-β family in wound healing, burns and scarring: a review. Int. J. Burns Trauma *2*, 18–28.
